# Dental Erosion

**DOI:** 10.1155/2012/356021

**Published:** 2012-12-05

**Authors:** Ana Carolina Magalhães, Annette Wiegand, Alix Young, Nadine Schlueter

**Affiliations:** ^1^Department of Biological Sciences, Bauru School of Dentistry, University of São Paulo, 17012-901 Bauru SP, Brazil; ^2^Clinic for Preventive Dentistry, Periodontology and Cariology, University of Zurich, 8032 Zurich, Switzerland; ^3^Department of Cariology and Gerodontology, Faculty of Dentistry, University of Oslo, 0455 Oslo, Norway; ^4^Department of Conservative and Preventive Dentistry, Dental Clinic, Justus Liebig University, 35392 Giessen, Germany

Dental erosion is a condition defined as tooth substance loss by exogenous or endogenous acids without bacterial involvement. The most important sources are dietary acids and gastric acid in patients with regurgitation and reflux disorders. Dental erosion is a multifactorial condition, where different chemical, biological, and behavioral factors contribute to its development. Progressive erosive loss may lead to functional and aesthetic limitations, as well as hypersensitivity, and extensive restorative treatment often becomes necessary.

As there are signs that the prevalence of erosion is increasing in several countries, research in the field of dental erosion has expanded over the last few years. In this special issue, we have invited investigators to contribute with original research as well as review manuscripts that will stimulate the continuing efforts in the understanding of the development of erosive lesions. Thirteen papers were submitted, from which 5 were finally accepted (approval rate: 38.5%). 

A. Mulic and coworkers present an investigation about dentists' general experience, knowledge about diagnosis, and treatment of dental erosive wear in young adults in Norway. They show that dentists are relatively up-to-date with respect to the clinical recording, diagnosis, and treatment of tooth erosive wear. However, dietary and salivary analyses were not given priority during the diagnosis process, and early and preventive treatment was lacking.

On the other hand, laboratory researchers have made concerted efforts to identify protective agents against erosion. Another paper tests if the in vitro application of fluoride (NaF), with or without hydroxyapatite (HAP), or casein phosphopeptide-amorphous calcium phosphate alone, were able to reduce early enamel erosion using nanohardness as the response variable. A. Z. Abdullah and coworkers demonstrate that 4500 mg/L fluoride, regardless of the presence of HAP, significantly increases the nanohardness of previously eroded enamel and reduces the progression of erosion.

However, not only the concentration, but also the type of fluoride salt might influence its protective effect against dental erosion. Accordingly, L. P. Comar and coworkers show a better in vitro effect of toothpastes containing TiF_4_ or SnF_2_ compared to NaF against enamel and dentin erosion and abrasion. These results are in accordance with previous studies showing that metal fluoride compounds performed better than conventional sodium fluoride in the prevention of tooth wear. 

A. B. Borges and coworkers raise the question of whether bleached enamel is more susceptible to erosive wear. They tested the effect of adding calcium and fluoride to 35% hydrogen peroxide (HP) bleaching gel on the susceptibility of enamel to erosion provoked by a soft drink in vitro. While the bleaching gel alone does not increase the enamel susceptibility to erosion, the addition of calcium gluconate to the bleaching gel even results in a reduced susceptibility of the enamel to extrinsic erosive challenges.

Finally, use of sealants as a minimally-invasive approach to prevent the progression of erosive lesions is analysed with special regard to the effect of light-curing time (F. J. Wegehaupt and coworkers). The in vitro results show that given a constant energy density, light-curing time does not have an influence on the permeability and stability of the sealants.

We hope that this special issue makes interesting reading, and that these studies will encourage further research on the diagnosis, prevention, and therapy of dental erosion.

*Ana Carolina Magalhães* *Ana Carolina Magalhães* *Annette Wiegand* *Annette Wiegand* *Alix Young* *Alix Young* *Nadine Schlueter* *Nadine Schlueter*

